# Weight Management Experiences Among People Affected by Overweight and Obesity Who Are Living With and Beyond Colorectal, Breast or Prostate Cancer: A Cross‐Sectional Survey

**DOI:** 10.1002/cam4.70885

**Published:** 2025-04-16

**Authors:** William Goodman, Phillipa Lally, Abigail Fisher, Rebecca J. Beeken

**Affiliations:** ^1^ Leeds Institute of Health Sciences University of Leeds Leeds UK; ^2^ School of Psychology University of Surrey Guildford UK; ^3^ Research Department of Behavioural Science and Health University College London London UK

**Keywords:** advice, breast cancer, cancer, colorectal cancer, obesity, overweight, prostate cancer, weight management

## Abstract

**Introduction:**

After a cancer diagnosis, recommendations for those living with and beyond cancer (LWBC) are to maintain a body mass index (BMI) < 25 and avoid weight gain. This study explored factors associated with receipt of and interest in weight management advice as well as engagement in weight management programmes (WMPs) among people LWBC affected by overweight and obesity.

**Methods:**

3456 participants living with or beyond breast, prostate or colorectal cancer and affected by overweight and obesity completed our questionnaire. Participants reported advice received on losing weight, interest in receiving weight management advice, enrolment in a lifestyle programme and their beliefs about maintaining a healthy weight to prevent cancer recurrence.

**Results:**

Logistic regression results suggested that those who received advice were more likely to be interested in advice (OR = 1.68; 95% CI = 1.30; 2.18) and to be enrolled in a WMP (OR = 1.60; 95% CI = 1.07; 2.40). The belief that maintaining a healthy weight could prevent cancer recurrence was associated with greater interest in weight management advice (OR = 1.47; 95% CI = 1.33; 1.62) and enrolment in a programme (OR = 1.57; 95% CI = 1.25; 1.99).

**Discussion:**

These results highlight that those who recall receiving weight management advice are more likely to be enrolled in a WMP, suggesting that advice should be offered by healthcare professionals to all those patients affected by overweight and obesity.

**Conclusions:**

This suggests that the receipt of weight management advice may encourage people LWBC who are affected by overweight and obesity to engage with WMPs.

## 1 Introduction

Cancer is one of the leading causes of mortality globally, with over 20 million newly diagnosed individuals and 9.7 million cancer‐related deaths in 2022 alone [1]. In 2020, breast, prostate and colorectal cancer were among the five most commonly reported cancers in the United Kingdom [2]. Recent advancements in our understanding of the mechanisms of the disease, technologies to support early detection and therapeutic strategies have led to dramatic improvements in cancer survival rates [3, 4, 5]. As a result, millions of patients are currently living beyond their cancer diagnosis, with a range of side effects from both their cancer and associated treatments.

There is an increasing body of evidence which suggests that obesity is associated with the development of 4%–8% of all cancers, including breast, colorectal and prostate [6]. Therefore, many individuals are likely to already have a higher weight at the time of diagnosis, which can be exacerbated by cancer treatments for which weight gain is a potential side effect. Studies have reported weight gain in 85% of women following a breast cancer diagnosis, 70% of men with a prostate cancer diagnosis and 76% of those with a colorectal cancer diagnosis [7, 8, 9]. Research in those living with and beyond breast cancer suggests that weight gain can be between 1 and 12 kg in 90% of the sample [10], greater than 5 kg in 27% of those living with and beyond colorectal cancer [11] and fat mass increased by 14% in those living with and beyond prostate cancer [12]. Excess weight is associated with unfavourable outcomes relating to cancer recurrence, overall mortality, and cancer‐specific mortality in breast, colorectal and prostate cancer [13, 14]. As a result, specialised guidelines issued by the governmental agencies and designated research councils recommend that those living with and beyond cancer (LWBC) maintain a healthy weight (body mass index [BMI] < 25) and avoid weight gain [15].

Weight management programmes (WMPs) can provide advice and support to the individuals, and some have been developed to address overweight and obesity in those LWBC. A systematic review of 20 weight loss intervention studies in 2028 women with breast cancer found low quality of evidence for a reduction in body weight, BMI and waist circumference and an increase in quality of life, with multimodal interventions (dietary change, exercise and psychosocial support) offering greater improvements compared to dietary aspects alone [16]. Conversely, among those living with and beyond prostate cancer, a systematic review that identified 20 randomised controlled trials (RCTs) of exercise and diet interventions found that diet interventions were more effective in weight reduction than exercise only interventions [17]. Overall, research suggests that WMPs appear to be acceptable for people LWBC who are affected by overweight and obesity, although our understanding is primarily restricted to the individuals that apply to take part in research studies. Therefore, we have limited understanding of engagement in WMPs among people LWBC in real‐world settings and whether they have received, or are interested in, advice on weight management.

Qualitative research suggests that those LWBC and affected by overweight and obesity are interested in receiving advice on weight management [18]. However, we do not know if this is true for all patients nor if certain demographic and clinical factors are associated with interest or receipt of advice. If interest differs among certain groups of individuals and/or advice is not being delivered to all who may benefit, this could highlight a need for targeted interventions at either the patient or healthcare professional (HCP) level.

Beliefs about the role that weight plays in the development of cancer could influence interest and engagement in WMPs post‐diagnosis. Cross‐sectional studies of the general population in the United Kingdom suggests that there is a poor understanding that maintaining a healthy body weight can help to reduce the risk of cancer development, although this awareness can be enhanced through public health campaigns [19, 20]. Qualitative research suggests that those LWBC are aware of the benefits of being physically active and that poor diet can increase the risk of cancer development [21, 22]. Furthermore, among those living with and beyond breast cancer, cross‐sectional research suggests that there is an awareness that being overweight is associated with cancer recurrence [23]. However, these studies do not provide us with an understanding as to whether these beliefs are associated with interest in weight management advice or engagement in WMPs.

Therefore, the current study aimed to explore the factors associated with receipt of/interest in weight management advice as well as engagement in existing WMPs among those affected by overweight and obesity.

## 2 Methods

### 2.1 Study Design and Sample

This study involved secondary analyses of cross‐sectional survey data from the “Health and Lifestyle After Cancer Questionnaire” [24]. The survey comprised of 10 sections covering: socio‐demographic and anthropometric variables; health; physical activity; diet and nutrition; alcohol; tobacco; lifestyle advice; beliefs about causes of cancer and recurrence; personal wellbeing; and enrolment in formal lifestyle programmes.

To be included in the study, participants had to be aged ≥ 18 years old, and must have received a primary diagnosis of breast, prostate or colorectal cancer. Participating hospitals were asked to send questionnaires to those diagnosed between 2012 and 2015 to help make the numbers manageable. Participants who were diagnosed outside of those dates but returned a completed questionnaire were still included within the current analyses. Therefore, participants were diagnosed with breast, prostate or colorectal cancer between 1994 and 2017. This study focused on a subsample of 3456 people that had a BMI greater than 25 kg/m^2^ and were therefore affected by overweight or obesity.

### 2.2 Procedure

The first page informed the participants that by completing and returning the questionnaire they were giving consent for their data to be used for research. Recruitment of survey participants was conducted through 10 collaborative NHS Trusts in London and Essex using the Clinical Research Network Portfolio. The questionnaires could be completed and returned to the research group either in a paper or an online format (an online link to the survey was provided on the invitation letters). Completed questionnaires were accepted between February 2015 and January 2018. Ethics approval was granted from South Central—Oxford B Committee of the NHS National Research Ethics Service Committees in December 2014 (reference number 14/SC/1369) [24].

### 2.3 Measures

Measures included in the questionnaire were designed for this study apart from the advice received and beliefs about weight and cancer measures, which were adapted from previous studies [25, 26]. Prior to the distribution of the questionnaire, it was piloted with two individuals LWBC (female and male) to ensure acceptability.

### 2.4 Demographic and Clinical Characteristics

Age was reported in years. Sex was reported as ‘male’ or ‘female’. Participants were asked to select their ethnic group from a list of 15 with an ‘other’ option which they could specify, these were condensed into a dichotomized variable of ‘white’ and ‘non‐white’, due to the low numbers of ‘non‐white’ respondents. Participants were asked about their marital status with options including married, single, divorced, separated and widowed; this was dichotomized into ‘married’ and ‘not married’ (including divorced, widowed, separated and single). Participants were asked to select their highest level of education achieved from ‘None’, ‘GCSE/vocational’ (equivalent to high school diplomas), ‘A‐Level’ (equivalent qualifications for entry into university), ‘Degree or above’ or ‘Other’; the participants that selected other had their responses reviewed by a member of the research team to assign their response to the corresponding level of the other listed education qualifications. This variable was treated as continuous for the analysis.

BMI was calculated from respondents reported height (cm) and weight (kg); kg/m^2^. BMI categories were derived from established cut‐offs of BMI (‘underweight’ below 18.5, ‘healthy weight’ 18.5–24.9, ‘overweight’ 25–29.9 and ‘obese’ above 30). These were then dichotomized into ‘underweight/healthy weight’ and ‘overweight/obese’. Participants were also asked to report the type of cancer they have been diagnosed (‘breast’, ‘prostate’ or ‘colorectal’), and the time since their most recent cancer diagnosis was calculated in months (in some cases an individual's most recent cancer diagnosis may not be breast, prostate or colorectal), from the date they reported being diagnosed and the date the questionnaire was received. Participants were asked to think about their most recent cancer diagnosis and whether this had spread to any other parts of their body, this was reported as either ‘yes’, ‘no’ or ‘don't know’ with ‘don't know’ treated as missing for the analysis. A treatment variable was created based upon the various treatment's respondents said they had received for their most recent cancer, the created groups were: ‘No treatment/active surveillance only’, ‘Surgery only’, ‘Surgery and at least one other treatment’ and ‘Any other combination of treatment’. Participants were asked whether they had any health problems from a list and asked to report any that were not listed, and from this a total number of comorbidities variable was created.

### 2.5 Beliefs About Weight and Cancer

Respondents were asked whether they believed maintaining a healthy weight was important for preventing cancer recurrence. Participants rated their belief on a scale from 1 to 5 (1 = ‘not at all important’ to 5 = ‘very important’).

### 2.6 Weight Management Advice and WMPs

Respondents were asked whether they had received any advice on losing weight from a HCP since their diagnosis (yes, no). They were also asked whether they were interested in receiving weight management advice from a HCP, rated from 1 to 4 (1 = ‘Not at all interested’ to 4 = ‘Very interested’). This was dichotomized into ‘Not at all interested’ (1) and ‘Interested’ (2–4).

Respondents were also asked whether they were currently enrolled in a lifestyle programme and to specify the name of the programme if they were. Responses to this question were classified as different types of programme, including WMPs. Responses classified as WMPs included Weight Watchers and Slimming World, which accounted for 87.0% of the WMPs (53.1% and 33.9%, respectively); other examples included Ace Lifestyle, Juice Plus and the 5‐2 Diet. The full table of WMPs can be found in Data S1.

### 2.7 Statistical Analysis

All analyses were performed using SPSS v26.0. Descriptive statistics were run on all variables included within the analyses and are presented for the full sample and those affected by overweight and obesity. Cancer type was included in descriptive statistics but was not included within the analyses due to multicollinearity with sex. Covariates included within the analyses were selected based on their associations in previous research regarding similar advice received and beliefs about cancer recurrence [27, 28, 29, 30].

Logistic regressions were run within those affected by overweight and obesity to determine the association between demographic and clinical characteristics and the dependent variables of advice received, interest in advice and current participation in a WMP. Belief about weight and cancer and advice received were also included in the regressions for interest in advice and membership of a WMP.

Little's MCAR test was run to determine the missingness of the data. The test was significant (*χ* ^2^[40, 3456] = 347.8, *p* < 0.001); therefore, the data were not missing completely at random. Multiple imputation was conducted to account for the missing data. There were five imputed datasets, and all variables that were included within the analyses were imputed and used as predictors for the imputation. The analyses presented in the paper are based on imputed data, and the completers analyses are presented in Data S3.

## 3 Results

### 3.1 Descriptive Statistics

A total of 13,500 questionnaires were sent and 5835 were returned (43% response rate). Descriptive statistics for the full sample and those affected by overweight and obesity (*n* = 3456; 59.2%) can be found in Table 1. Descriptive statistics stratified by cancer type can be found in Data S2.

**TABLE 1:** Descriptive statistics for the full sample and those with overweight and obesity.

Variables	Respondents full sample (*N* = 5835)	Respondents overweight and obese (*N* = 3456)
Age		
Mean (SD)	67.4 (11.8)	67.3 (10.8)
Missing, *N*	36	15
Sex, *N* (%)		
Male	2552 (43.9)	1667 (48.3)
Female	3266 (56.1)	1781 (51.7)
Missing	16	8
Highest education, *N* (%)		
None	1709 (32.3)	1078 (34.4)
GCSE/vocational	1613 (30.5)	997 (31.8)
A‐level	584 (11.1)	332 (10.6)
Degree or above	1379 (26.1)	724 (23.1)
Missing	550	325
Marital status, *N* (%)		
Married	4037 (69.4)	2447 (70.9)
Divorced/separated/widowed/single	1781 (30.6)	1003 (29.1)
Missing	17	6
Ethnicity—dichotomised, *N* (%)		
White	5249 (90.5)	3136 (91.0)
Other	554 (9.5)	309 (9.0)
Missing	32	11
BMI		
Mean (SD)	27.0 (4.9)	29.6 (4.2)
Missing, *N*	336	0
Cancer type, *N* (%)		
Breast	2786 (47.8)	1536 (44.4)
Prostate	1839 (31.5)	1206 (34.9)
Colorectal	1210 (20.7)	714 (20.7)
Missing	0	0
Time since recent cancer diagnosis (months)		
Mean (SD)	36.1 (13.9)	35.2 (13.3)
Missing, *N*	36	20
Cancer spread, *N* (%)		
Yes	558 (11.0)	342 (11.4)
No	4498 (89.0)	2647 (88.6)
Missing	779	467
Treatment, *N* (%)		
No treatment/active surveillance only	296 (5.1)	163 (4.8)
Surgery only	1081 (18.8)	652 (19.2)
Surgery and at least one other treatment	2967 (51.6)	1704 (50.0)
Any other combination of treatment	1411 (24.5)	886 (26.0)
Missing	80	51
Total comorbidities		
Mean (SD)	1.3 (1.3)	1.3 (1.3)
Missing, *N* (%)	0	0
Advice received—losing weight, *N* (%)		
Yes	948 (18.4)	810 (26.1)
No	4209 (81.6)	2291 (73.9)
Missing	678	355
Belief in maintaining a healthy weight is associated with preventing cancer recurrence, *N* (%)		
1 (not at all important)	277 (5.5)	159 (5.3)
2	162 (3.2)	105 (3.5)
3	695 (13.8)	451 (15.0)
4	1005 (20.0)	648 (21.5)
5 (very important)	2893 (57.5)	1647 (54.7)
Missing	803	446
Interest in weight management advice, *N* (%)		
Yes	3734 (75.4)	2463 (80.0)
No	1221 (24.6)	616 (20.0)
Missing	880	377
Member of a weight management programme, *N* (%)		
Yes	164 (2.9)	130 (3.8)
No	5595 (97.1)	3288 (96.2)
Missing	76	38

Abbreviations: BMI, body mass index; *N*, number of participants; SD, standard deviation.

### 3.2 Weight Management Advice and Membership of a WMP

Among those affected by overweight and obesity, older participants were less likely to have received advice, less likely to be interested in advice and less likely to be enrolled in a WMP (Table 2). Females were more likely to be enrolled in a WMP compared to males (OR = 8.19, 95% CI = 4.29; 15.61). Additionally, non‐white individuals were more likely to have received advice (OR = 2.43, 95% CI = 1.74; 3.40) and more likely to be interested in receiving advice (OR = 1.68, 95% CI = 1.09; 2.59) but less likely to be enrolled in a WMP (OR = 0.19, 95% CI = 0.07; 0.55). Participants with higher BMIs were more likely to have received advice (OR = 1.21, 95% CI = 1.18; 1.24) and to be a member of a WMP (OR = 1.05, 95% CI = 1.01; 1.08) but did not appear to be more interested in advice. Participants who had received advice on weight management from a HCP and who believed that maintaining a healthy weight is associated with cancer recurrence were more likely to both be interested in advice (advice: OR = 1.73, 95% CI = 1.30; 2.32; and belief: OR = 1.46, 95% CI = 1.35; 1.58) and to be enrolled in a WMP (advice: OR = 1.57, 95% CI = 1.05; 2.35; and belief: OR = 1.50, 95% CI = 1.04; 2.18). Results from the completers analyses in Data S3 are consistent with the imputed results presented in Table 2. Results from analyses stratified by cancer type can be found in Data S4.

**TABLE 2:** Logistic regressions—factors associated with advice received, interest in advice and attending weight management programmes (*n* = 3456).

Variables	Advice received—losing weight (reference: no)	Interest in weight management advice (reference: no)	Member of a weight management programme (reference: no)
	OR (95% CI)	OR (95% CI)	OR (95% CI)
Age	**0.99** (**0.98**; **1.00**)	**0.96** (**0.95**; **0.97**)	**0.98** (**0.96**; **1.00**)
Sex (reference: male)	**0.72** (**0.55**; **0.93**)	**0.74** (**0.58**; **0.94**)	**8.19** (**4.29**; **15.61**)
Highest education	1.08 (0.99; 1.17)	**1.12** (**1.02**; **1.23**)	0.88 (0.72; 1.08)
Marital status (reference: married)	**0.81** (**0.66**; **0.99**)	0.87 (0.71; 1.06)	0.73 (0.48; 1.10)
Ethnicity (reference: white)	**2.43** (**1.74**; **3.40**)	**1.68** (**1.09**; **2.59**)	**0.19** (**0.07**; **0.55**)
BMI	**1.21** (**1.18**; **1.24**)	1.02 (0.99; 1.05)	**1.05** (**1.01**; **1.08**)
Time since recent cancer diagnosis (months)	**1.01** (**1.00**; **1.02**)	1.00 (0.99; 1.01)	1.01 (1.00; 1.03)
Cancer spread (reference: no)	**0.60** (**0.44**; **0.81**)	0.99 (0.67; 1.46)	0.98 (0.55; 1.75)
Treatment (reference: no treatment)			
Surgery only	1.47 (0.93; 2.33)	0.67 (0.41; 1.08)	1.82 (0.23; 14.32)
Surgery and one other treatment	1.20 (0.75; 1.90)	0.85 (0.54; 1.34)	1.74 (0.23; 13.24)
Any combination of other treatment	1.36 (0.88; 2.10)	0.79 (0.50; 1.25)	2.40 (0.31; 18.46)
Number of comorbidities	**1.18** (**1.10**; **1.26**)	1.05 (0.97; 1.14)	0.94 (0.81; 1.10)
Advice received—losing weight (reference: no)		**1.73** (**1.30**; **2.32**)	**1.57** (**1.05**; **2.35**)
Belief in maintaining a healthy weight is associated with preventing cancer recurrence		**1.46** (**1.35**; **1.58**)	**1.50** (**1.04**; **2.18**)

*Note:* Bold values are statistically significant at *p* < 0.05.

## 4 Discussion

In this sample of people, LWBC 59.2% were affected by overweight and obesity, of whom only 23.4% had received advice about weight management. The majority of those affected by overweight and obesity (71.3%) were interested in weight management advice, but just 3.8% were currently enrolled in a WMP. Those who were younger were more likely to receive and be interested in advice and be enrolled in a WMP. Those who were not of white ethnicity were more likely to receive and be interested in advice but were less likely to be enrolled in a WMP. Importantly, those who had received advice or who believed that maintaining a healthy weight prevents cancer recurrence were also more interested in advice and more likely to be engaged in a WMP.

This study identified that only a small proportion of those LWBC affected by overweight and obesity were enrolled on a WMP (3.8%), although 71% were interested in receiving weight management advice. This aligns with a recent study which has indicated that a majority of those LWBC affected by overweight and obesity perceive they need to lose weight, suggesting that either there may be barriers to accessing current programmes or that they do not meet the needs of this group [31]. The most commonly reported programmes were Slimming World and Weight Watchers, which, as they are targeted towards the general population, would have a lack of tailored support for those LWBC. Future research should further explore the experiences of people LWBC who use existing WMPs that are widely available. Understanding individuals' experiences of these programmes and adapting and tailoring these to people LWBC may prove more cost effective than development of new WMPs. Working alongside those LWBC will be crucial for ensuring the success of any planned adaptations, as previous studies utilising co‐creation have been able to identify key barriers to uptake such as a lack of advisors with the knowledge of the physical and emotional issues faced by those LWBC [32].

Of those affected by overweight and obesity, only 23.4% reported receiving advice on losing weight from a HCP, despite obesity being associated with the development of 4%–8% of all cancers and associated with cancer recurrence and mortality [6, 13, 14]. This is in line with previous cross‐sectional research among clinicians (*N* = 323) working in colorectal cancer, which found that only 19% rated their knowledge of weight management recommendations as good, compared with 41% who rated this as poor, and 50% wanted additional training in this area [26]. Our study found that receiving advice on weight management was associated with interest in weight management advice and being a member of a WMP. This suggests that receiving advice does not discourage wanting to receive further advice and may act as a prompt to action for engaging in WMPs. However, it may also suggest that the advice received was not sufficient. Further work is needed to ensure that any advice delivered is done so consistently to all that might benefit, given that those who were younger, male, and of white ethnicity were both more likely to receive weight management advice and to be interested in further advice. Oncologists have limited dietary training and awareness of lifestyle guidelines, and thus may have difficulty integrating weight management in general cancer care [33, 34]. Previous research also suggests that HCPs do not believe that they are the appropriate individuals to be having these discussions with patients, although patients report wanting to receive this advice from them [21, 22, 35]. Therefore, training is needed to support HCPs in providing weight management advice that could benefit patients.

This study found that close to half of the sample believed maintaining a healthy weight to prevent cancer recurrence is very important. Previous qualitative research has found that patients are aware of the link between diet and physical activity and the development of cancer, and to a lesser extent associations with cancer recurrence [21, 22]. Previous quantitative research among a representative sample of the English population found that more than 60% of adults believed being overweight is related to cancer development, whereas less than 40% believed that diet and physical activity were related [36]. In the current study, the belief that maintaining a healthy weight can prevent cancer recurrence was also associated with interest in receiving advice on losing weight and being a member of a WMP. This suggests it may be helpful to provide this information to people LWBC to increase awareness and potentially encourage receptivity to weight management advice and support.

However, the way in which this information is provided to those LWBC needs to be considered. For example, this information could lead to feelings of body‐related guilt and shame that some people LWBC already hold, and thus the role of weight in cancer should be discussed sensitively with patients [37]. HCPs have reported an awareness of these issues but that they require strategies in being able to broach this subject in an appropriate manner with their patients [38]. Previous research also suggests that consideration needs to be given as to when the most suitable time for this advice is provided so as not to cause additional distress to those LWBC [38]. Research suggests that those living with and beyond breast cancer may have established concerns regarding their weight which could overshadow their cancer diagnosis [39]. This suggests a more appropriate time for this advice to be delivered may be after their primary cancer treatment; however, there is growing interest in the potential for weight management support pre‐treatment to help improve outcomes [40].

The findings from this study suggests that certain groups of people are more likely to be enrolled in a WMP. Females are more likely to be enrolled in a WMP, which is in line with research in the general population looking at commercially available WMPs [41]. This could reflect societal pressures and WMPs appealing more to females [42]. Although interventions have been designed for males LWBC within research studies [43], commercially available WMPs may need to address male engagement issues. Similarly, those who were not of white ethnicity were more likely to be interested in advice but less likely to be enrolled in a WMP, which could suggests that these programmes need cultural adaptations to ensure they are inclusive. HCPs could also provide more direction to locally available WMPs to encourage uptake from all groups.

This study has several limitations. Firstly, this is a cross‐sectional analysis; therefore, we cannot determine causality, for which longitudinal data with a prospective study design would be needed. Furthermore, as this is a secondary analysis, certain measures were not optimised for this research question. For example, the wording of the question for WMPs only identifies those that are currently on a WMP, not those that may have been on one in the near past or at another time post‐diagnosis. BMI was based on self‐report data, which may underestimate people's weight and/or overestimate height [44]. Furthermore, the reporting of advice that was received is dependent on participant recall; therefore, we may not have received an accurate overview of advice received. The findings may also not be generalisable to the wider population due to selection bias, as 90% of the sample is of white ethnicity, whereas the general population in England and Wales is 81.7% white [45]. Previous research has identified underrepresentation of ethnic minorities in health research [46] and although we do not have data on the socio‐economic status of the sample, research also suggests that those that respond to health surveys are generally from higher socio‐economic status backgrounds [47].

In conclusion, this study has identified that among people LWBC affected by overweight and obesity, less than a quarter recalled receiving advice on losing weight despite most being interested in receiving this advice. Positively, those that had received advice and were aware of the role weight plays in cancer recurrence were more interested in weight management advice and more likely to be enrolled in a WMP. However, overall, less than 5% of those affected by overweight and obesity reported enrolment in these programmes. This highlights a clear need for oncology HCPs to provide additional support to all patients affected by excess weight and to ensure that WMP are acceptable to and meeting the needs of those LWBC. Information about the role of excess weight in cancer may encourage individuals to engage with such programmes, but further research is needed to ensure this is delivered in a sensitive, non‐stigmatising way to the underrepresented groups.

## Author Contributions

**William Goodman:** writing – review and editing, writing – original draft, formal analysis, data curation. **Phillipa Lally:** writing – review and editing, data curation, project administration. **Abigail Fisher:** writing – review and editing, funding acquisition, conceptualization. **Rebecca J. Beeken:** writing – review and editing, supervision, funding acquisition, conceptualization, methodology.

## Disclosure

Authorship declaration: All authors met each of the following four criteria to qualify for authorship: (1) have made substantial contributions to conception and design, or acquisition of data or analysis and interpretation of data; (2) been involved in drafting the manuscript or revising it critically for important intellectual content; (3) given final approval of the version to be published. Each author should have participated sufficiently in the work to take public responsibility for appropriate portions of the content; and (4) agreed to be accountable for all aspects of the work in ensuring that questions related to the accuracy or integrity of any part of the work are appropriately investigated and resolved.

## Ethics Statement

Ethical approval was obtained through the National Research Ethics Service Committee South Central—Oxford B (reference number 14/SC/1369). The following statement was provided on the questionnaire: ‘By completing this questionnaire you are consenting to your anonymous information being used for research on lifestyle in people diagnosed with cancer’. The study was performed in accordance with the Declaration of Helsinki.

## Consent

Participants were made aware in the consent form that their anonymized data would be used in journal publications.

## Conflicts of Interest

The authors declare no conflicts of interest.

## Supporting information


Data S1.

Data S2.

Data S3.

Data S4.

## Data Availability

The data sets generated during and/or analysed during this study are available from the corresponding author on reasonable request.
