# Supplementary material for: Weight Management Experiences Among People Affected by Overweight and Obesity Who Are Living With and Beyond Colorectal, Breast or Prostate Cancer: A Cross‐Sectional Survey
Source: Cancer Med. 2025 Apr 16;14(8):e70885. doi: 10.1002/cam4.70885 (PMC12001425; doi:10.1002/cam4.70885)
Supplement: Supplementary file 1 — Data S1. [file CAM4-14-e70885-s004.docx]

**Data S1: Weight management programmes that participants were currently enrolled on (N=130)**

| **Weight Management Programme** | **Count N (%)** |
| --- | --- |
| Slimming World | 69 (53.1) |
| Weight Watchers | 44 (33.9) |
| Ace Lifestyle | 3 (2.3) |
| Herbalife | 2 (1.5) |
| More Life | 2 (1.5) |
| Juice Plus | 2 (1.5) |
| Sainsbury’s Diet Plan | 1 (0.8) |
| Glow Diet | 1 (0.8) |
| NHS Health Trainer Programme | 1 (0.8) |
| Dietician Local NHS | 1 (0.8) |
| Cambridge Weight Loss Plan | 1 (0.8) |
| 5-2 Diet | 1 (0.8) |
| FitGirlSlim | 1 (0.8) |
| NHS My Weight Matters | 1 (0.8) |
