# Supplementary material for: Weight Management Experiences Among People Affected by Overweight and Obesity Who Are Living With and Beyond Colorectal, Breast or Prostate Cancer: A Cross‐Sectional Survey
Source: Cancer Med. 2025 Apr 16;14(8):e70885. doi: 10.1002/cam4.70885 (PMC12001425; doi:10.1002/cam4.70885)
Supplement: Supplementary file 2 — Data S2. [file CAM4-14-e70885-s002.docx]

**Data S2: Descriptive statistics stratified by cancer type**

| **Variables** | **Breast cancer (N=1536)** | **Prostate cancer (N=1206)** | **Colorectal cancer (N=714)** |
| --- | --- | --- | --- |
| **Age** |  |  |  |
| Mean (SD) | 63.0 (11.5) | 71.3 (7.5) | 69.7 (10.8) |
| Missing N (%) | 3 | 8 | 4 |
| **Sex** **N(%)** |  |  |  |
| Male | 16 (1.0) | 1201 (99.6) | 450 (63.0) |
| Female | 1519 (98.9) | 0 (0) | 262 (36.7) |
| Missing | 1 (0.1) | 5 (0.4) | 2 (0.3) |
| **Highest education N(%)** |  |  |  |
| None | 370 (24.1) | 448 (37.1) | 260 (36.4) |
| GCSE/Vocational | 517 (33.7) | 278 (23.1) | 202 (28.3) |
| A-Level | 181 (11.8) | 92 (7.6) | 59 (8.3) |
| Degree or above | 369 (24.0) | 219 (18.2) | 136 (19.0) |
| Missing | 99 (6.4) | 169 (14.0) | 57 (8.0) |
| **Marital status N(%)** |  |  |  |
| Married | 989 (64.4) | 946 (78.4) | 512 (71.7) |
| Divorced/Separated/Widowed/Single | 547 (35.6) | 255 (21.1) | 201 (28.2) |
| Missing | 0 | 5 (0.4) | 1 (0.1) |
| **Ethnicity – dichotomised N(%)** |  |  |  |
| White | 1362 (88.7) | 1091 (90.5) | 683 (95.7) |
| Other | 170 (11.1) | 109 (9.0) | 30 (4.2) |
| Missing | 4 (0.3) | 6 (0.5) | 1 (0.1) |
| **BMI** |  |  |  |
| Mean (SD) | 30.1 (4.6) | 29.0 (3.5) | 29.7 (4.0) |
| Missing N (%) | 0 | 0 | 0 |
| **Time since recent cancer diagnosis (months)** |  |  |  |
| Mean (SD) | 34.9 (11.0) | 36.7 (15.6) | 33.4 (13.1) |
| Missing N(%) | 1 | 16 | 3 |
| **Cancer spread N(%)** |  |  |  |
| Yes | 1204 (78.4) | 905 (75.0) | 538 (75.4) |
| No | 152 (9.9) | 105 (8.7) | 85 (11.9) |
| Missing | 180 (11.7) | 196 (16.3) | 91 (12.7) |
| **Treatment N(%)** |  |  |  |
| No treatment/active surveillance only | 5 (0.3) | 140 (11.6) | 18 (2.5) |
| Surgery only | 113 (7.4) | 245 (20.3) | 294 (41.2) |
| Surgery and at least one other treatment | 1261 (82.1) | 114 (9.5) | 329 (46.1) |
| Any other combination of treatment | 136 (8.9) | 686 (56.9) | 64 (9.0) |
| Missing | 21 (1.4) | 21 (1.7) | 9 (1.3) |
| **Total comorbidities** |  |  |  |
| Mean (SD) | 1.3 (1.3) | 1.3 (1.2) | 1.4 (1.4) |
| Missing N(%) | 0 | 0 | 0 |
| **Advice received – Losing weight N(%)** |  |  |  |
| Yes | 356 (23.2) | 281 (23.3) | 173 (24.2) |
| No | 1043 (67.9) | 794 (65.8) | 454 (63.6) |
| Missing | 137 (8.9) | 131 (10.9) | 87 (12.2) |
| **Belief in maintaining a healthy weight is associated with preventing cancer recurrence N(%)** |  |  |  |
| 1 Not at all important | 53 (3.5) | 85 (7.0) | 21 (2.9) |
| 2 | 50 (3.3) | 30 (2.5) | 25 (3.5) |
| 3 | 209 (13.6) | 152 (12.6) | 90 (12.6) |
| 4 | 277 (18.0) | 239 (19.8) | 132 (18.5) |
| 5 Very important | 789 (51.4) | 508 (42.1) | 350 (49.0) |
| Missing | 158 (10.3) | 192 (15.9) | 96 (13.4) |
| **Interest in weight management advice N(%)** |  |  |  |
| Yes | 1151 (74.9) | 839 (69.6) | 473 (66.2) |
| No | 258 (16.8) | 215 (17.8) | 143 (20.0) |
| Missing | 127 (8.3) | 152 (12.6) | 98 (13.7) |
| **Member of a weight management programme N(%)** |  |  |  |
| Yes | 102 (6.6) | 8 (0.7) | 20 (2.8) |
| No | 1417 (92.3) | 1183 (98.1) | 688 (96.4) |
| Missing | 17 (1.1) | 15 (1.2) | 6 (0.8) |
