# Supplementary material for: Weight Management Experiences Among People Affected by Overweight and Obesity Who Are Living With and Beyond Colorectal, Breast or Prostate Cancer: A Cross‐Sectional Survey
Source: Cancer Med. 2025 Apr 16;14(8):e70885. doi: 10.1002/cam4.70885 (PMC12001425; doi:10.1002/cam4.70885)
Supplement: Supplementary file 3 — Data S3. [file CAM4-14-e70885-s003.docx]

**Data S3: Completers logistic regressions - Factors associated with advice received, interest in advice and weight management programmes**

| Variables | Advice received (reference: no; n=2393)  OR (95%CI) | Interest in advice (reference: no; n=2010)  OR (95%CI) | Member of a weight management programme (reference: no; n=2151)  OR (95%CI) |
| --- | --- | --- | --- |
| Age | **0.99 (0.98; 1.00)** | **0.97 (0.96; 0.99)** | **0.98 (0.96; 1.00)** |
| Sex (reference: male) | **0.72 (0.55; 0.93)** | **0.60 (0.43; 0.83)** | **5.62 (2.65; 11.92)** |
| Education | 1.09 (0.99; 1.19) | **1.16 (1.03; 1.30)** | 0.82 (0.66; 1.01) |
| Marital status (reference: married) | **0.78 (0.62; 0.98)** | 0.96 (0.72; 1.27) | 0.76 (0.47; 1.23) |
| Ethnicity (reference: white) | **2.31 (1.67; 3.18)** | **2.84 (1.46; 5.53)** | **0.28 (0.10; 0.78)** |
| BMI | **1.22 (1.19; 1.25)** | 1.02 (0.99; 1.06) | 1.03 (0.98; 1.07) |
| Time since cancer diagnosis | **1.01 (1.00; 1.02)** | 1.00 (0.99; 1.01) | 1.02 (0.99; 1.03) |
| Cancer spread (reference: no) | **0.66 (0.47; 0.93)** | 1.04 (0.68; 1.59) | 1.11 (0.58; 2.14) |
| Treatment (reference: no treatment) |  |  |  |
| Surgery only | 1.68 (0.96; 2.95) | 0.57 (0.27; 1.19) | 1.14 (0.14; 9.29) |
| Surgery and one other treatment | 1.32 (0.75; 2.34) | 0.86 (0.41; 1.81) | 1.25 (0.16; 9.83) |
| Any combination of other treatment | 1.34 (0.77; 2.34) | 0.60 (0.29; 1.24) | 1.28 (0.16; 10.30) |
| Number of comorbidities | **1.21 (1.12; 1.31)** | 1.06 (0.95; 1.17) | 1.01 (0.85; 1.20) |
| Advice received (reference: no) |  | **1.65 (1.18; 2.31)** | 1.53 (0.96; 2.44) |
| Belief in maintaining a healthy weight is associated with preventing cancer reoccurrence |  | **1.45 (1.32; 1.60)** | **1.50 (1.16; 1.92)** |

Note: where 1.0 is bold this remains a significant result and is the result of rounding.
