# Supplementary material for: Weight Management Experiences Among People Affected by Overweight and Obesity Who Are Living With and Beyond Colorectal, Breast or Prostate Cancer: A Cross‐Sectional Survey
Source: Cancer Med. 2025 Apr 16;14(8):e70885. doi: 10.1002/cam4.70885 (PMC12001425; doi:10.1002/cam4.70885)
Supplement: Supplementary file 4 — Data S4. [file CAM4-14-e70885-s001.docx]

**Data S4**

**Logistic regressions - Factors associated with advice received, interest in advice and attending weight management programmes (Colorectal cancer n=714)**

| Variables | Advice received – Losing weight (reference: no)  OR (95%CI) | Interest in weight management advice (reference: no)  OR (95%CI) | Member of a weight management programme (reference: no)  OR (95%CI) |
| --- | --- | --- | --- |
| Age | 0.99 (0.97;1.01) | **0.96 (0.93;0.98)** | 0.99 (0.95;1.04) |
| Sex (reference: male) | 0.74 (0.47;1.16) | 0.81 (0.53;1.24) | **5.79 (1.96;17.14)** |
| Highest education | 1.08 (0.89;1.31) | 1.11 (0.92;1.34) | 1.04 (0.67;1.63) |
| Marital status (reference: married) | 0.87 (0.55;1.37) | 0.87 (0.56;1.31) | 0.92 (0.33;2.56) |
| Ethnicity (reference: white) | 1.92 (0.81;4.54) | 0.88 (0.32;2.46) | - |
| BMI | **1.26 (1.19;1.33)** | 0.99 (0.93;1.05) | 1.09 (1.00;1.19) |
| Time since recent cancer diagnosis (months) | **1.01 (1.00;1.03)** | 0.99 (0.98;1.01) | 1.03 (1.00;1.07) |
| Cancer spread (reference: no) | **0.44 (0.23;0.86)** | 1.01 (0.50;2.01) | - |
| Treatment (reference: no treatment) |  |  |  |
| Surgery only | 1.87 (0.35;10.11) | 0.73 (0.21;2.55) | 0.52 (0.05;4.99) |
| Surgery and one other treatment | 1.61 (0.31;8.33) | 0.87 (0.25;3.06) | 0.50 (0.05;4.91) |
| Any combination of other treatment | 2.18 (0.39;12.08) | 0.82 (0.17;3.91) | 0.66 (0.05;9.24) |
| Number of comorbidities | **1.23 (1.06;1.43)** | 0.98 (0.83;1.16) | 0.82 (0.54;1.23) |
| Advice received – Losing weight (reference: no) |  | **2.66 (1.52;4.67)** | 1.30 (0.45;3.79) |
| Belief in maintaining a healthy weight is associated with preventing cancer recurrence |  | **1.34 (1.06;1.68)** | 1.22 (0.70;2.15) |

**Logistic regressions - Factors associated with advice received, interest in advice and attending weight management programmes (Prostate cancer n=1206)**

| Variables | Advice received – Losing weight (reference: no)  OR (95%CI) | Interest in weight management advice (reference: no)  OR (95%CI) | Member of a weight management programme (reference: no)  OR (95%CI) |
| --- | --- | --- | --- |
| Age | 1.00 (0.98;1.02) | **0.96 (0.94;0.99)** | 0.98 (0.88;1.09) |
| Highest education | 1.09 (0.94;1.25) | **1.18 (1.01;1.37)** | 0.55 (0.14;2.14) |
| Marital status (reference: married) | 0.76 (0.51;1.12) | 1.09 (0.73;1.61) | - |
| Ethnicity (reference: white) | **3.33 (1.83;6.06)** | 1.68 (0.82;3.42) | - |
| BMI | **1.32 (1.26;1.40)** | **1.06 (1.00;1.12)** | 1.13 (0.96;1.34) |
| Time since recent cancer diagnosis (months) | **1.01 (1.00;1.03)** | 1.01 (0.99;1.02) | 1.02 (0.98;1.07) |
| Cancer spread (reference: no) | **0.52 (0.28;0.96)** | 0.94 (0.38;2.30) | 1.83 (0.32;10.70) |
| Treatment (reference: no treatment) |  |  |  |
| Surgery only | 1.56 (0.87;2.78) | 0.63 (0.35;1.15) | - |
| Surgery and one other treatment | 0.83 (0.40;1.73) | 0.69 (0.35;1.38) | - |
| Any combination of other treatment | 1.28 (0.77;2.14) | 0.72 (0.42;1.25) | - |
| Number of comorbidities | **1.21 (1.06;1.38)** | 1.10 (0.96;1.26) | 0.79 (0.40;1.56) |
| Advice received – Losing weight (reference: no) |  | 1.64 (0.91;2.95) | 0.97 (0.17;5.50) |
| Belief in maintaining a healthy weight is associated with preventing cancer recurrence |  | **1.50 (1.34;1.67)** | 1.45 (0.62;3.38) |

**Logistic regressions - Factors associated with advice received, interest in advice and attending weight management programmes (Breast cancer n=1536)**

| Variables | Advice received – Losing weight (reference: no)  OR (95%CI) | Interest in weight management advice (reference: no)  OR (95%CI) | Member of a weight management programme (reference: no)  OR (95%CI) |
| --- | --- | --- | --- |
| Age | **0.98 (0.97;1.00)** | 0.96 (0.95;0.98) | **0.97 (0.95;1.00)** |
| Highest education | 1.08 (0.95;1.23) | 1.07 (0.93;1.24) | 0.86 (0.69;1.07) |
| Marital status (reference: married) | 0.83 (0.63;1.10) | 0.77 (0.57;1.03) | 0.71 (0.44;1.14) |
| Ethnicity (reference: white) | **2.16 (1.44;3.25)** | **2.19 (1.12;4.28)** | **0.21 (0.08;0.61)** |
| BMI | **1.15 (1.12;1.19)** | 1.02 (0.98;1.05) | 1.03 (0.99;1.08) |
| Time since recent cancer diagnosis (months) | 1.01 (1.00;1.02) | 1.00 (0.99;1.02) | 1.01 (0.99;1.03) |
| Cancer spread (reference: no) | 0.69 (0.42;1.13) | 1.05 (0.64;1.74) | 1.09 (0.58;2.07) |
| Treatment (reference: no treatment) |  |  |  |
| Surgery only | - | 3.49 (0.43;28.56) | - |
| Surgery and one other treatment | - | 4.24 (0.55;32.53) | - |
| Any combination of other treatment | - | 3.15 (0.42;23.77) | - |
| Number of comorbidities | **1.14 (1.02;1.27)** | 1.06 (0.94;1.20) | 0.99 (0.83;1.18) |
| Advice received – Losing weight (reference: no) |  | 1.43 (0.91;2.25) | **1.71 (1.08;2.72)** |
| Belief in maintaining a healthy weight is associated with preventing cancer recurrence |  | **1.50 (1.31;1.72)** | 1.56 (0.99;2.48) |

| **Variables in the Equation** | | | | | | | | | | | | | |
| --- | --- | --- | --- | --- | --- | --- | --- | --- | --- | --- | --- | --- | --- |
| Imputation Number | | | B | S.E. | Wald | df | Sig. | Exp(B) | 95% C.I.for EXP(B) | | Fraction Missing Info. | Relative Increase Variance | Relative Efficiency |
|  |  |  |  |  |  |  |  |  | Lower | Upper |  |  |  |
| Pooled | Step 1^a^ | Age (yrs) | -.026 | .011 |  |  | .019 | .974 | .954 | .996 | .024 | .024 | .995 |
|  |  | Sex(1) | .198 | 1.061 |  |  | .852 | 1.219 | .152 | 9.747 | .003 | .003 | .999 |
|  |  | Highest_Education | -.149 | .110 |  |  | .177 | .861 | .693 | 1.070 | .126 | .136 | .975 |
|  |  | Marital_Status(1) | -.343 | .241 |  |  | .155 | .710 | .443 | 1.139 | .024 | .025 | .995 |
|  |  | Ethnicity_Dichotomy(1) | -1.543 | .535 |  |  | .004 | .214 | .075 | .610 | .013 | .013 | .997 |
|  |  | BMI | .030 | .022 |  |  | .178 | 1.030 | .987 | 1.075 | .056 | .058 | .989 |
|  |  | Time Since Recent Cancer Date (Months) | .006 | .010 |  |  | .551 | 1.006 | .987 | 1.025 | .008 | .008 | .998 |
|  |  | Cancer spread(1) | .089 | .325 |  |  | .785 | 1.093 | .577 | 2.069 | .043 | .044 | .992 |
|  |  | Treatment(1) | 18.490 | 17303.863 |  |  | .999 | 107176662.992 | .000 | . | .000 | .000 | 1.000 |
|  |  | Treatment(2) | 18.340 | 17303.863 |  |  | .999 | 92204994.589 | .000 | . | .000 | .000 | 1.000 |
|  |  | Treatment(3) | 18.679 | 17303.863 |  |  | .999 | 129498016.130 | .000 | . | .000 | .000 | 1.000 |
|  |  | Total comorbidities (inc. number of other conditions) | -.010 | .089 |  |  | .915 | .991 | .832 | 1.180 | .020 | .021 | .996 |
|  |  | Advice received - Losing weight(1) | .536 | .236 |  |  | .023 | 1.709 | 1.076 | 2.715 | .036 | .036 | .993 |
|  |  | Cancer recurrence - Trying to maintain a healthy weight | .447 | .208 |  |  | .056 | 1.563 | .986 | 2.478 | .672 | 1.597 | .882 |
|  |  | Constant | -22.271 | 17303.863 |  |  | .999 | .000 | .000 | . | .000 | .000 | 1.000 |
| a. Variable(s) entered on step 1: Age (yrs), Sex, Highest_Education, Marital_Status, Ethnicity_Dichotomy, BMI, Time Since Recent Cancer Date (Months), Cancer spread, Treatment, Total comorbidities (inc. number of other conditions), Advice received - Losing weight, Cancer recurrence - Trying to maintain a healthy weight. | | | | | | | | | | | | | |
